# Impact of *Cocos nucifera* oil mouthwash on salivary glycoproteins: A randomized trial

**DOI:** 10.1016/j.jobcr.2026.101444

**Published:** 2026-03-28

**Authors:** Maithili Muthamma KM, Sreelakshmi S, Nishmitha N. Hegde, Chaithra Lakshmi V, Harshitha Somanatha, Mithra N. Hegde

**Affiliations:** a Department of Conservative Dentistry and Endodontics, AB Shetty Memorial Institute of Dental Sciences, Nitte (Deemed to be University), Deralakatte, Mangaluru, 5750018, Karnataka, India; b AB Shetty Memorial Institute of Dental Sciences, Nitte (Deemed to be University), Deralakatte, Mangaluru, 575018, India

**Keywords:** *Cocos nucifera* oil, Salivary glycoproteins, Dental caries, Amine fluoride, Mouthwash, Randomized trial

## Abstract

**Background:**

Salivary glycoproteins contribute to enamel protection, lubrication, and microbial regulation, making them useful biochemical indicators of oral health.

**Objective:**

To evaluate the impact of *Cocos nucifera* oil (CNO) mouthwash on total salivary protein and selected salivary glycoproteins (MUC5B, albumin, globulin, and amylase) in adults with moderate caries over 28 days.

**Methods:**

In this double-blind, parallel-group randomized trial, 40 adults aged 18–40 years (DMFT >3) were assigned to use either CNO mouthwash (10 mL, 10 min daily) or amine fluoride (AF) mouthwash (15 mL, 1 min daily) for 28 days. Unstimulated saliva was collected at baseline and day 28 to assess total protein, MUC5B, albumin, globulin, and amylase. Salivary glycoproteins and total salivary protein may reflect different aspects of oral biochemical defence and should be interpreted independently. Statistical analysis was performed using IBM SPSS version 23.0. Within-group comparisons were conducted using the Wilcoxon signed-rank test, while between-group comparisons were performed using the Mann–Whitney *U* test. Statistical significance was set at p ≤ 0.05.

**Results:**

Both mouthwashes produced modest biochemical changes after 28 days. CNO resulted in a statistically significant increase in total salivary protein compared with amine fluoride (p = 0.035). No significant between-group differences were observed for MUC5B, albumin, globulin, or amylase.

**Conclusion:**

CNO mouthwash produced a modest increase in total salivary protein; however, specific glycoproteins showed no significant changes. These results provide preliminary biochemical evidence only, and no clinical inferences regarding caries prevention or oral defense can be made. Larger trials with clinical endpoints are required.

## 1 Introduction

Dental caries remains the most prevalent oral disease worldwide and results from a complex interaction between oral microbiota, dietary habits, and host factors. Alongside etiological factors, the natural protective mechanisms of the oral environment particularly saliva play a critical role in maintaining oral health and preventing demineralization. Saliva contains numerous proteins and glycoproteins that contribute to lubrication, enamel protection, microbial regulation, and formation of the acquired enamel pellicle.

Salivary biomarkers have been widely explored as indicators of caries susceptibility. Among these, glycoproteins such as mucins, albumin, globulin, and amylase are of particular interest because they are directly involved in the protective functions of saliva. Alterations in these components may reflect changes in the biochemical defence capacity of the oral environment. Importantly, total salivary protein represents a composite measure and does not necessarily indicate changes in specific protective glycoproteins.^1^

Preventive dentistry increasingly focuses on interventions that reduce microbial load and enhance natural defence mechanisms. Amine fluoride, an organically derived compound with well-established anti-cariogenic and remineralizing properties, is widely used for chemical plaque control.^2^ Amflor® (Group Pharmaceuticals Ltd, India), an amine fluoride-based mouthwash, has demonstrated effectiveness in reducing plaque accumulation and caries risk.^3^ However, its influence on salivary glycoprotein profiles has not been clearly elucidated.

Traditional practices such as oil pulling with virgin coconut oil (*Cocos nucifera* oil; CNO) have also gained attention due to their reported antimicrobial and anti-inflammatory properties. Studies have shown that CNO can reduce salivary bacterial counts and improve plaque and gingival indices. Nevertheless, existing evidence primarily focuses on microbiological and clinical outcomes, while its effect on specific salivary glycoproteins remains unclear.^4^

Thus, despite evidence supporting the antimicrobial benefits of both amine fluoride and coconut oil, **their potential influence on the biochemical protective protein composition of saliva remains poorly understood.** This represents an important knowledge gap, as modulation of salivary glycoproteins may reflect changes in oral defence beyond conventional clinical indices.

Therefore, the present randomized clinical trial was designed to evaluate and compare the effects of *Cocos nucifera* oil and amine fluoride mouthwashes on total salivary protein and selected salivary glycoproteins over a 28-day period. This distinction is essential to avoid overgeneralization, as changes in total protein do not necessarily indicate modulation of specific protective glycoproteins involved in oral defence.

## 2 Materials and methods

The study was conducted in a dental educational and research institute and was reviewed and approved by the Institutional Ethical Committee (ETHICS/242/2022). The study was registered in the Clinical Trial Registry of India (CTRI) (Reg. number: CTRI/2022/10/046334). The sample size calculation was based on the results of a study by Kaushik M et al., which analyzed the change in salivary *S. mutans* levels after coconut oil pulling, with a 5% level of significance and 80% power. This indicated that 18 participants per group were required. Considering a 10% expected attrition rate, 20 participants were included per group, resulting in a total of 40 participants.

A total of 40 participants aged 18–40 years, with a minimum of 20 intact teeth and a DMFT score >3, visiting the OPD pool of the department between September 2023 and October 2023, were enrolled. Participants allergic to coconut oil or fluoride, with xerostomia, oral ulcers, edentulism, or medically compromised conditions (e.g., immunosuppression, radiotherapy, diabetes), or systemic/local diseases affecting salivary secretion were excluded. Two experienced and calibrated investigators conducted participant screening. Written informed consent was obtained from all participants after a full explanation of the study objectives. Any identified dental needs were addressed before inclusion.

**Fig. 1:**
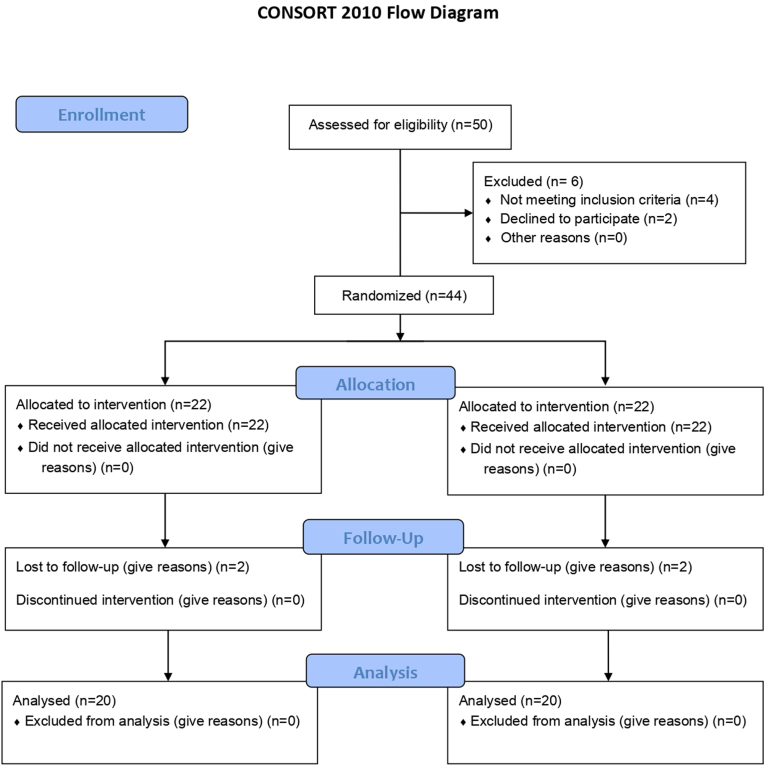
Study design- CONSORT Flow chart.

The study adhered to the CONSORT guidelines (2010), ensuring a rigorous and standardized approach to design, conduct, analysis, and reporting. The study design is summarized in Fig. 1.

### 2.1 Study design

This was a randomized, parallel-group clinical trial with a 1:1 allocation ratio. Participants were randomly assigned to the CNO mouthwash (experimental group) and amine fluoride mouthwash (control group).

### 2.2 Blinding

Both mouthwashes were provided in identical, coded, and unlabelled containers. Participants and outcome assessors were blinded to group allocation. Although participants could potentially identify the mouthwash by taste or texture**,** this did not influence the outcome measurements. Therefore, the study is considered double-blinded.

### 2.3 Randomization

Two independent investigators performed randomization**.** The first investigator generated the random allocation sequence and maintained allocation concealment using sequentially numbered, opaque, sealed envelopes. This investigator also enrolled participants but remained blinded to group assignments. The second investigator opened the envelopes in sequence and assigned participants to their respective groups without access to other clinical details beyond eligibility criteria. This procedure ensured adequate allocation concealment and minimized selection bias.

A total of 40 participants were randomized equally into two groups (n = 20 per group). All randomized participants completed the 28 days intervention and follow-up saliva collection. As there were no dropouts or protocol deviations, the final analysis included all randomized participants. The analysis therefore followed a per-protocol approach, which was equivalent to intention-to-treat analysis in this trial due to complete participant retention.

### 2.4 Saliva collection method

Unstimulated saliva samples (5 mL) were collected via the passive drool method between 10:00–11:00 a.m. Participants were instructed to abstain from eating, drinking, brushing, smoking, or using mouthwash for at least 2 h prior. Volunteers sat upright with their heads forward.^5^

### 2.5 Laboratory procedures

Saliva samples were centrifuged at 2500 rpm for 10 min, after which the supernatant was collected and stored at −80 °C (New Brunswick, UK) until analysis. Salivary glycoproteins were quantified using standardized biochemical methods. MUC5B levels were measured using a sandwich ELISA kit (KRISHGEN BioSystems, India), with absorbance recorded at 450 nm and results expressed in ng/mL. Total salivary proteins were estimated by the Biuret method (AGAPPE Diagnostics Ltd, India) at 546 nm and expressed in g/dL. Albumin concentration was determined using the bromocresol green dye method with absorbance at 630 nm, also expressed in g/dL. Globulin levels were calculated by subtracting the albumin value from the total protein. Salivary amylase activity was assessed using the AGAPPE kit by measuring the change in optical density per minute (ΔOD/min) at 405 nm, and results were expressed in U/L.

These methods provide reliable assessment of pre- and post-intervention salivary glycoprotein levels.

### 2.6 Interventions

Group A used 10 mL of *Cocos nucifera* oil (Swadeshi Desha, India), rinsed for 10 min each morning after brushing for 28 days, allowing sufficient emulsification and prolonged contact with oral surfaces. Group B used 15 mL of an amine fluoride–based mouthwash, Amflor® (Group Pharmaceuticals Ltd, India), rinsed for 1 min after brushing for the same 28-day period; the higher volume and shorter rinsing time align with the mouthwash's rapid anti-cariogenic action.

Participants were instructed not to consume anything for 30 min after rinsing. Amine fluoride was selected as the comparator due to its well-established anti-cariogenic properties, providing a standard reference. Participant adherence was monitored through daily logs and follow-up reminders, ensuring compliance with the prescribed mouthwash regimen and enhancing the reliability of the collected data. Follow-up saliva samples were collected after 28 days for analysis of MUC5B, albumin, amylase, globulin, and total protein.

Participant adherence to the prescribed mouthwash regimen was assessed using daily compliance logs and periodic follow-up reminders. All participants returned completed logs at the end of the study, indicating full compliance with the intervention protocol. No missed doses or deviations from the rinsing instructions were reported.

### 2.7 Outcome measures

The primary outcome of the study was the change in total salivary protein following the 28-day intervention. The secondary outcome was the assessment of changes in specific salivary glycoproteins (MUC5B, albumin, globulin, and amylase) and comparison of these changes between the *Cocos nucifera* oil (CNO) and amine fluoride groups.

### 2.8 Adverse events monitoring

Participants were instructed to report any discomfort, allergic reactions, mucosal irritation, burning sensation, altered taste, or any other untoward effects throughout the study period. At each follow-up interaction, participants were specifically questioned regarding possible adverse events. No adverse events or side effects were reported in either group during the 28-day intervention period.

### 2.9 Statistical analysis

Although appropriate non-parametric tests (Wilcoxon signed-rank and Mann–Whitney U tests) were used for within- and between-group comparisons, important statistical limitations must be acknowledged when interpreting the findings. The sample size calculation for this trial was derived from a previous study evaluating changes in salivary *S. mutans* counts following coconut oil pulling. As this outcome differs substantially from the multiple biochemical endpoints assessed in the present study, the trial was not specifically powered to detect differences in salivary glycoproteins. Consequently, the study may be underpowered for these biochemical outcomes.

Furthermore, multiple salivary biomarkers (total protein, MUC5B, albumin, globulin, and amylase) were analyzed without adjustment for multiplicity. In this context, the statistically significant between-group difference observed for total salivary protein (p = 0.035) should be interpreted cautiously, as the possibility of a type I error cannot be excluded. This borderline p-value, in the absence of multiplicity correction, does not constitute strong statistical evidence of a true effect.

In addition, effect sizes and confidence intervals were not calculated, which limits the ability to assess the magnitude and precision of the observed differences. Reliance solely on p-values restricts the interpretability and clinical relevance of the findings.

These statistical considerations indicate that the results should be viewed as exploratory and hypothesis-generating rather than confirmatory. Future studies should perform outcome-specific sample size estimation, apply appropriate corrections for multiple comparisons, and report effect sizes with confidence intervals to strengthen the robustness and interpretability of the results.

## 3 Results

Of the 40 participants randomized (20 in each group), all completed the study and were included in the final analysis. There were no losses to follow-up, exclusions, or protocol deviations. Consequently, outcome data were available for all participants originally allocated to each group. A total of 40 participants aged 18–40 years with a minimum of 20 intact teeth and a DMFT score >3 was enrolled from the department OPD between September 2023 and October 2023. Fig. 1 presents the CONSORT flow diagram for participant recruitment and allocation.

Table 1 presents the baseline demographic and oral health characteristics of the study participants. The mean age of participants was 27.05 ± 6.13 years in the amine fluoride (AF) group and 28.12 ± 5.27 years in the *Cocos nucifera* oil (CNO) group. The mean salivary pH values were comparable between the groups (6.88 ± 0.52 in AF and 6.92 ± 0.58 in CNO). Oral hygiene status assessed using OHI-S was also similar, with mean scores of 0.70 ± 0.38 in the AF group and 0.68 ± 0.30 in the CNO group, indicating that participants were well matched at baseline.

**Table 1:** Baseline background of the study participants.

Variables	AF (n = 20) Mean ± SD	CNO (n = 20) Mean ± SD
**Age (Years)**	27.05 ± 6.13	28.12 ± 5.27
**pH**	6.88 ± 0.52	6.92 ± 0.58
**OHI-S**	0.70 ± 0.38	0.68 ± 0.3

AF, Amine Fluoride, CNO, *Cocos nucifera* oil, OHI-S, Simplified Oral Hygiene Index, values are expressed in Mean ± SD.

**Fig. 2:**
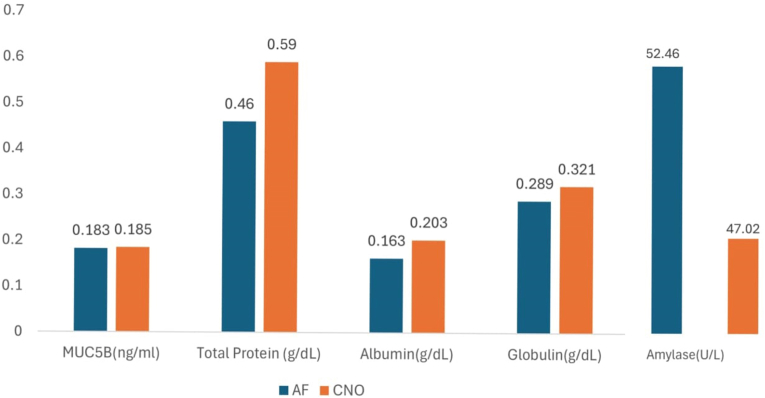
Comparison of pre-usage and post-usage values of MUC5B, STP, Albumin, Globulin and Amylase.

Post-intervention analysis of salivary biomarkers (Table 2) demonstrated that within-group comparisons using the Wilcoxon signed-rank test showed a statistically significant increase in total salivary protein in the CNO group after intervention (p = 0.007), while no significant change was observed in the AF group (p = 0.398). A statistically significant change in albumin was observed in the AF group (p = 0.013), whereas no significant change was seen in the CNO group (p = 0.455). Similarly, amylase levels showed a significant reduction in the AF group (p = 0.015) but not in the CNO group (p = 0.097). Globulin levels showed a significant increase in the CNO group (p = 0.009) but not in the AF group (p = 0.096). No statistically significant changes were observed for MUC5B in either group (p > 0.05). Fig. 2.

**Table 2:** Comparison of pre and post-usage values of salivary biomarkers between the two mouthwash groups.

Salivary biomarkers	Pre-Usage		Post usage				Difference (Post usage- Pre usage)			Effect size (r)	95% CI	
	AF	CNO	AF	CNO	Wilcoxon Signed rank test		AF	CNO	Mann–Whitney *U* test		Lower bound	Upper bound
	Mean	Mean	Mean	Mean	AF	CNO	Mean	Mean	p-value			
MUC5B(ng/ml)	0.1805	0.1855	0.1830	0.1850	0.848	0.718	0.0025	0.0005	0.500	0.031	−0.10	0.10
Total Protein (g/dL)	0.4350	0.3735	0.4600	0.5900	0.398	0.007	0.0250	0.2165	0.752	0.3	−0.240	0.020
Albumin(g/dL)	0.2090	0.1635	0.1990	0.2035	0.013	0.455	0.0455	0.0045	0.343	0.232	−0.90	0.10
Globulin(g/dL)	0.2265	0.2890	0.1740	0.3215	0.096	0.009	0.0625	0.1475	0.751	0.196	−0.150	0.070
Amylase(U/L)	38.2275	52.4685	37.9605	47.0285	0.015	0.097	14.19	9.0670	0.752	0.117	−9.530	19.070

∗p < 0.05 Statistically Significant, p > 0.05 Non-Significant, NS.

AF, Amine Fluoride; CNO, *Cocos nucifera* oil; CI, Confidence Interval.

Between-group comparisons of the change scores (post-usage minus pre-usage) using the Mann–Whitney *U* test revealed no statistically significant differences between the AF and CNO groups for MUC5B (p = 0.500), total protein (p = 0.752), albumin (p = 0.343), globulin (p = 0.751), or amylase (p = 0.752). The 95% confidence intervals for all biomarkers crossed zero, further indicating the absence of significant between-group differences. Effect sizes (r) were small for all comparisons.

Compliance with the mouthwash regimen was 100% in both groups based on participant logs and follow-up verification. No adverse events were reported by any participant in either group during the study period.

## 4 Discussion

Saliva plays a central role in oral homeostasis through proteins and glycoproteins that contribute to enamel protection, lubrication, and microbial regulation.^6^, ^7^, ^8^ In this randomized, double-blind, parallel-group trial, no statistically significant between-group differences were observed in total salivary protein, MUC5B, albumin, globulin, or amylase after 28 days of mouthwash use.

Within-group analysis demonstrated a statistically significant increase in total salivary protein and globulin levels in the CNO group, while the AF group showed significant changes in albumin and amylase levels. However, these changes were not reflected in the between-group comparisons of change scores, and the 95% confidence intervals for all biomarkers crossed zero, indicating the absence of a true differential effect between the two mouthwashes. Effect sizes were small for all comparisons.

Since total salivary protein represents a composite biochemical measure rather than a functionally specific biomarker, the observed within-group increase in the CNO group does not indicate selective modulation of protective salivary pathways. Similarly, the changes observed in albumin, globulin, and amylase within individual groups should be interpreted cautiously, as they were not sufficiently large to produce meaningful differences when compared between groups.

Importantly, the study did not include microbiological assessments, plaque indices, gingival parameters, or caries-related clinical outcomes. Therefore, the observed biochemical variations cannot be directly linked to improved oral defence, reduced cariogenic potential, or clinical benefit. These findings should be regarded as exploratory biochemical observations that may inform future hypothesis-driven research rather than evidence of therapeutic efficacy.

The minimal or inconsistent changes in individual glycoproteins may be attributable to the short intervention period (28 days), during which substantial shifts in gland-regulated salivary proteins are unlikely. Individual variability in salivary composition, hydration status, diet, and oral microbiota may also have limited the magnitude of detectable changes.

Previous studies on both amine fluoride and coconut oil have primarily reported microbiological and clinical improvements, such as reductions in plaque and salivary bacterial counts. In contrast, their influence on the biochemical protein composition of saliva has received limited attention. Mechanistically, CNO may enhance interaction of bioactive components with oral surfaces through emulsification, and lauric acid has been suggested to form compounds with antimicrobial potential. However, such explanations remain speculative in the absence of microbiological or plaque assessments in the present study.^9^

The significant within-group changes observed in the AF group for albumin and amylase are consistent with its established anti-cariogenic and remineralizing properties, supporting its role as a clinically relevant comparator in the present trial.^10^ However, the absence of significant between-group differences indicates that these changes did not translate into superiority over CNO in terms of salivary biochemical modulation.

This study has several methodological strengths, including prior ethical approval, prospective registration in the Clinical Trial Registry of India, and a randomized parallel-group design with allocation concealment and blinding of participants and outcome assessors. Standardized protocols for saliva collection, timing, storage, and biochemical analysis minimized pre-analytical and analytical variability. The use of a clinically relevant comparator and adherence monitoring further strengthened internal validity and the reliability of the biochemical measurements.

Limitations include the small sample size, restricted age range (18-40 years), short intervention duration, partial participant blinding due to sensory differences between mouthwashes, and absence of clinical or microbiological outcome measures, which limit generalizability and clinical interpretation.

An important additional limitation relates to the statistical framework of the study. The sample size was calculated based on changes in salivary *S. mutans* counts from previous literature rather than on the multiple biochemical endpoints assessed here. Consequently, the study was not specifically powered to detect meaningful differences in salivary glycoproteins, increasing the likelihood of type II error.

Furthermore, multiple salivary biomarkers were analyzed without adjustment for multiple comparisons. In this context, the statistically significant within-group findings must be interpreted cautiously, as the possibility of type I error cannot be excluded. These statistical considerations further reinforce that the findings should be interpreted as exploratory rather than confirmatory.

Future studies should include larger and more diverse populations, longer follow-up periods, and integration of clinical, microbiological, and salivary endpoints such as plaque index, gingival health, microbial profiling, and caries incidence to determine whether CNO mouthwash produces measurable and clinically meaningful oral health benefits.

## 5 Conclusion

Within the limitations of this study, both CNO and amine fluoride mouthwashes produced minor biochemical variations in salivary composition over 28 days. Although a modest increase in total salivary protein was observed in the CNO group, no significant changes were noted in specific salivary glycoproteins. In the absence of microbiological or clinical outcome measures, these findings should be interpreted as exploratory biochemical observations with uncertain clinical implications.

Further well-powered studies incorporating clinical, microbiological, and salivary endpoints are necessary to determine whether CNO mouthwash offers measurable benefits for oral health.

## Ethics approval and consent to participate

This study was conducted in accordance with relevant guidelines and regulations. The study was approved by the Institutional Ethics Committee (ETHICS/242/2022) and registered in the Clinical Trial Registry (CTRI) (Registration number: CTRI/2022/10/046334). All participants provided informed consent prior to completing the study.

## Author contributions

Dr Maithili Muthamma KM: Contributed to conception, design, data acquisition and interpretation, drafted and critically revised the manuscript.

Dr Sreelakshmi S: Contributed to conception, design, data acquisition and interpretation, drafted and critically revised the manuscript.

Dr Nishmitha N Hegde: Contributed to conception, design, data acquisition and interpretation, drafted and critically revised the manuscript.

Chaithra Lakshmi V: Contributed to conception, design, data acquisition and interpretation, drafted and critically revised the manuscript.

Harshitha Somanatha: Contributed to conception, design, data acquisition and interpretation, drafted and critically revised the manuscript.

Prof Dr Mithra N Hegde: Contributed to conception, design, data acquisition and interpretation, drafted and critically revised the manuscript.

“All authors gave their final approval and agree to be accountable for all aspects of the work”.

## Funding statement

There was no external financial support for the study.

## Declaration of competing interest

None.
